# Screening and Characterization of Potentially Suppressive Soils against *Gaeumannomyces graminis* under Extensive Wheat Cropping by Chilean Indigenous Communities

**DOI:** 10.3389/fmicb.2017.01552

**Published:** 2017-08-15

**Authors:** Paola Durán, Milko Jorquera, Sharon Viscardi, Victor J. Carrion, María de la Luz Mora, María J. Pozo

**Affiliations:** ^1^Scientific and Technological Bioresource Nucleus, Universidad de La Frontera Temuco, Chile; ^2^Biocontrol Research Laboratory, Universidad de La Frontera Temuco, Chile; ^3^Applied Microbial Ecology Laboratory, Department of Chemical Sciences and Natural Resources, Universidad de La Frontera Temuco, Chile; ^4^Netherlands Institute of Ecology, (NIOO-KNAW) Wageningen, Netherlands; ^5^Department of Soil Microbiology and Symbiotic Systems, Estación Experimental del Zaidín (CSIC) Granada, Spain

**Keywords:** *Gaeumannomyces graminis*, Mapuche, microbial communities, suppressive soils, *Triticum aestivum*, *take-all disease*, biocontrol

## Abstract

Wheat production around the world is severely compromised by the occurrence of “take-all” disease, which is caused by the soil-borne pathogen *Gaeumannomyces graminis* var. tritici (Ggt). In this context, suppressive soils are those environments in which plants comparatively suffer less soil-borne pathogen diseases than expected, owing to native soil microorganism activities. In southern Chile, where 85% of the national cereal production takes place, several studies have suggested the existence of suppressive soils under extensive wheat cropping. Thus, this study aimed to screen Ggt-suppressive soil occurrence in 16 locations managed by indigenous “Mapuche” communities, using extensive wheat cropping for more than 10 years. Ggt growth inhibition *in vitro* screenings allowed the identification of nine putative suppressive soils. Six of these soils, including Andisols and Ultisols, were confirmed to be suppressive, since they reduced take-all disease in wheat plants growing under greenhouse conditions. Suppressiveness was lost upon soil sterilization, and recovered by adding 1% of the natural soil, hence confirming that suppressiveness was closely associated to the soil microbiome community composition. Our results demonstrate that long-term extensive wheat cropping, established by small Mapuche communities, can generate suppressive soils that can be used as effective microorganism sources for take-all disease biocontrol. Accordingly, suppressive soil identification and characterization are key steps for the development of environmentally-friendly and efficient biotechnological applications for soil-borne disease control.

## Introduction

Currently, the main concerns in modern agriculture are those associated with the effect of climate change on biotic and abiotic parameters in arable soils, and their impact on crop yields and food supply at global level (Soussana et al., 2010). In this context, several authors have pointed out an increase of soil-borne disease incidence in winter cereals as a consequence of climate change (French et al., 2009; Manici et al., 2014). Similarly, these studies have also described that climate change may contribute to certain soil-borne pathogen migration toward niches or regions previously uncolonized by these pathogens (French et al., 2009).

The southern region of Chile produces around 85% of cereals, where 40% is wheat (*Triticum aestivum* L.; ODEPA, 2016). However, wheat production is frequently reduced by the incidence of “take-all” disease, which is caused by the soil-borne pathogen *Gaeumannomyces graminis* var tritici (Ggt; Andrade et al., 2011), causing the highest wheat crop losses in Chile (Moya-Elizondo et al., 2015). Soil-borne pathogen incidence in cereal cropping is difficult to control due to their natural persistence in soils and the inefficiency of chemical controls (De Coninck et al., 2015); thus, biological control becomes a very promising alternative to prevent soil diseases. The incidence of take-all disease outbreak is favored under particular soil conditions, called “conducive” soils (Chng et al., 2015). In contrast, “suppressive” soils occur as a natural phenomena, preventing soil-borne pathogen establishment or reducing disease incidence (Jara et al., 2011) even in the presence of a susceptible host plant and favorable soil or climate conditions. In this context, several studies have shown that native soil microorganism activity can be pivotal in Ggt disease suppression (Weller et al., 2002; Cook, 2003; Mendes et al., 2011, 2013). Ggt (and other soil-borne pathogens) suppressive soils have been reported and characterized around the world (Bull et al., 1991; Bithell et al., 2012; Chng et al., 2013).

In the twentieth century, agrarian policies resulted in the establishment of numerous small farmers practicing extensive agriculture in southern Chile, particularly in the “Region de La Araucania” (38°54′00″S; 72°40′00″S) (Clapp, 1998). Most farmers belong to the “Mapuche” ethnic group, which represents 50% of the total population in La Araucania. The Mapuche community is characterized by the use of ancestral agronomic techniques to produce their own agricultural products without the application of chemicals such as, commercial fertilizers and pesticides. The extensive Mapuche agriculture is mainly directed to family group subsistence. Under this scenario, we hypothesize that this long-term land-use could represent a natural and effective source of suppressive soils against soil-borne pathogen diseases. Thus, suppressive soils may also be relevant in the context of alterations related to soil-borne pathogen incidence and migration that have been predicted by climate change, and the deleterious effect on intensive agro-chemical product use in arable soils (Meza and Silva, 2009; Neuenschwander, 2010). In fact, Andrade et al. (2011) detected five Ggt-suppressive soils located in La Araucania with a long history of monoculture and natural pasture. Similarly, Arismendi et al. (2012) reported the presence of *Pseudomonas fluorescens* strains, which are able to produce 2, 4-DAPG, a known biocontrol compound involved in soil Ggt suppression (Mavrodi et al., 2012; Weller et al., 2012), in 13 soils from La Araucania and Los Lagos regions. Moreover, we have recently isolated and characterized four endophytic bacteria from wheat plants in this area (*Acinetobacter* sp. E6.2, *Bacillus* sp. E8.1, *Bacillus* sp. E5 and *Klebsiella* sp. E1), which are able to inhibit Ggt mycelia growth *in vitro*, promote plant growth, and diminish take-all disease incidence under greenhouse experiments (Durán et al., 2014). Although most studies on Ggt suppression have focussed on bacteria, there are some reports showing that some fungal strains can also reduce Ggt incidence (Macia-Vicente et al., 2008). These examples illustrate the great potential of native microorganisms as soil inoculants able to increase plant growth and prevent soil-borne pathogen disease incidence in cereal cropping. Despite of this potential, studies focusing on Ggt-suppressive soils occurrence, and their characterization, are still very limited.

The main objective of our study was to screen Ggt-suppressive soils in 16 locations managed by indigenous Mapuche communities from La Araucania, southern Chile, where extensive wheat cropping was applied for more than 10 years. Soil chemical properties and microbial community composition were characterized. Furthermore, soils were categorized as Ggt suppressive or conducive based on their potential for pathogen inhibition *in vitro*, and their efficacy in controlling take-all disease under greenhouse conditions.

## Materials and methods

### Soil sampling

**Figure 1:**
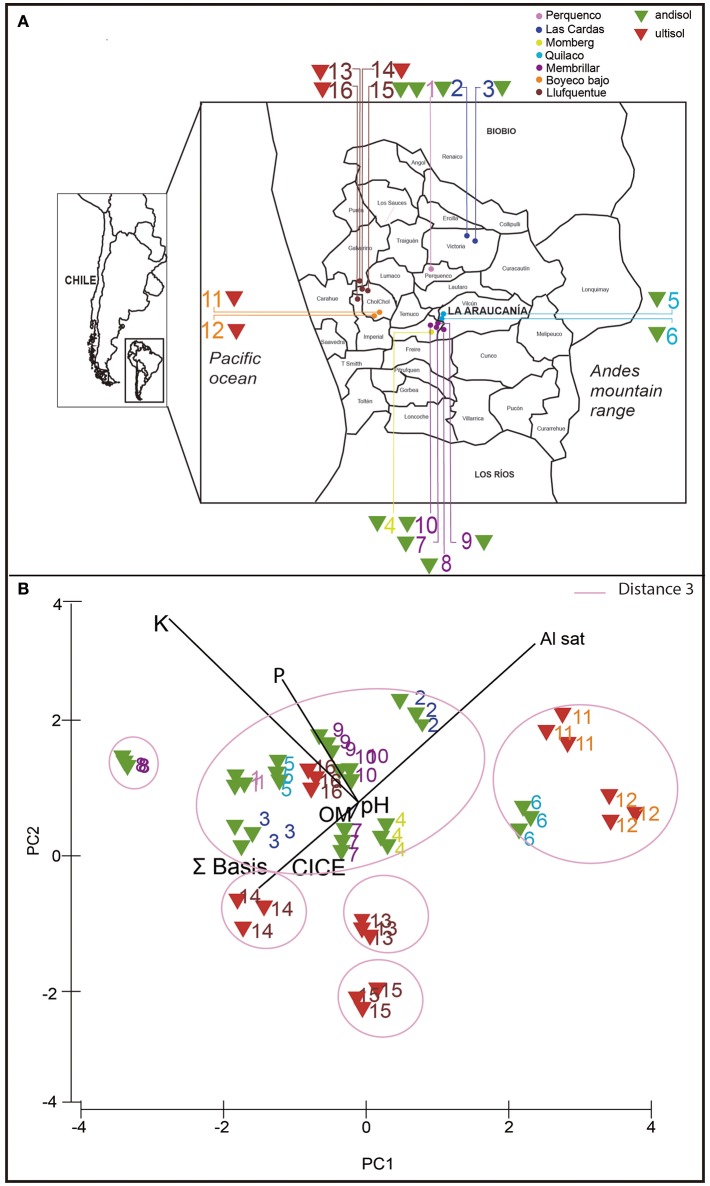
Sampling locations **(A)** and principal component analysis (PCA) analysis **(B)** based on chemical properties of 16 rhizosphere soils used in this study. Triangle colors indicate type of soil: andisol (green) and ultisol (red).

In southern Chile, acidic volcanic soils (Andisols and Ultisols) are the predominant soil types supporting the bulk of agricultural and forestry production. Andisols include modern and recent ash deposits, and Ultisols correspond to ancient deposits (Andrade et al., 2011). Soil samples were collected from extensive wheat cropping areas managed by Mapuche communities in 16 locations from La Araucania (Figure 1A; Table 1). Nine samples (number 3, 4, 5, 7, 10, 12, 13, 14, 15, and 16) were taken from soils with a long rotation history between wheat monoculture and natural pasture for more than 10 years, and six samples (number 2, 6, 8, 9, 11, and 12) were taken from soils with wheat monoculture including oat rotation. An additional sample number 1 was taken from a Ggt-conducive soil with wheat, including clover rotation, and used as positive control. Parent material from soils 1 to 10 was classified as Andisol, whereas 11–16 were classified as Ultisols.

**Table 1:** Sample locations and cropping history of soils used in this study.

**Soil**	**Parent material**	**Location**	**GPS coordinates**	**Cropping history**
1	Andisol	Perquenco	38°22′25.8″S 72°23′07.5″W	Conducive soil (control)
2	Andisol	Las Cardas-1	38°19′09.3″S 72°17′03.9″W	Wheat- triticale- oat (more than 10 years)
3	Andisol	Las Cardas-2	38°19′32.7″S 72°17′18.6″W	Wheat (10 years)- natural pasture (10 years)-wheat
4	Andisol	Momberg	38°50′30.5″S 72°32′21.4″W	Natural pasture (10 years)-clover- wheat-wheat
5	Andisol	Quilaco-1	38°43′25.9″S 72°27′19.2″W	Natural pasture (10 years)-wheat
6	Andisol	Quilaco-2	38°43′35.7″S 72°27′34.5″W	Clover (5 years)-oat- wheat
7	Andisol	Membrillar-1	38°45′01.6″S 72°28′28.4″W	Natural pasture-wheat (15 years)
8	Andisol	Membrillar-2	38°45′15.1″S 72°26′63.2″W	Oat-wheat-wheat (30 years)
9	Andisol	Membrillar-3	38°44′57.9″S 72°28′58.7″W	Wheat-oat+natural pasture
10	Andisol	Membrillar-4	38°44′52.8″S 72°29′21.3″W	Natural pasture (10 years)- wheat (2 years)
11	Ultisol	Boyeco bajo-1	38°39′00.4″S 72°41′41.7″W	Wheat-oat-wheat-wheat (more than 20 years)
12	Ultisol	Boyeco bajo-2	38°39′48.8″S 72°42′08.8″W	Natural pasture (10 years)- wheat- oat- wheat
13	Ultisol	Llufquentue-1	38°25′44.5″S 72°46′12.3″W	Wheat- wheat- natural pasture- wheat (more than 10 years)
14	Ultisol	Llufquentue-2	38°28′55.1″S 72°43′25.6″W	Wheat (more than 20 years)
15	Ultisol	Llufquentue-3	38°28′53.1″S 72°43′27.6″W	Wheat (more than 20 years)
16	Ultisol	Llufquentue-4	38°29′38.2″S 72°45′26.2″W	Natural pasture (10 years)- wheat- wheat

Samples were collected from rhizosphere and bulk soil at 0–20 cm depth, and then stored in a cold room at 5°C until usage. Soil sample chemical properties were determined as follow. Briefly, available P (P_Olsen_) was extracted by using 0.5 M Na-bicarbonate and analyzed by using the molybdate method (Murphy and Riley, 1962). Organic matter contents were estimated by wet digestion (Walkley and Black, 1934). Soil pH was measured in 1:2.5 soil/deionized water suspensions. Exchangeable potassium (K^+^), calcium (Ca^2+^), magnesium (Mg^2+^), and sodium (Na^+^) were extracted with 1 M ammonium acetate (CH_3_COONH_4_) at pH 7.0 and analyzed by flame atomic adsorption spectrophotometry (FAAS) (Warncke and Brown, 1998). Exchangeable aluminum (Al^3+^) was extracted with 1 M KCl and analyzed by FAAS (Bertsch and Bloom, 1996). All samples analyses were made in triplicate. To group and determine significant differences between samples based on their chemical properties, data were imported into the PRIMER 7 software (PRIMER-E Ltd, Ivybridge, UK), transformed and normalized using square-root followed by a log (Xþ1) transformations (Lee et al., 2012). Then, a distance matrix was generated based on Euclidean distances and samples were grouped by hierarchical clustering (group average), and then visualized by principal component analysis (PCA).

### Bacterial community composition and Ggt detection in soil samples

Bacterial community compositions in rhizosphere and bulk soil samples were examined by denaturing gradient gel electrophoresis (DGGE), according to Iwamoto et al. (2000). Total genomic DNA was extracted from 0.5 to 1 g of soil samples using the PowerSoil® DNA Isolation Kit (Mo-Bio Laboratories, Carlsbad, CA, USA), according to manufacturer's instructions. The 16S rRNA gene fragments were amplified by touchdown PCR, using EUBf933-GC/EUBr1387 primer set (Iwamoto et al., 2000). DGGE analysis was performed using a DCode system (Bio-Rad Laboratories, Inc., USA). The PCR product (20 μL) was loaded onto a 6% (v/v) polyacrylamide gel with a 40–70% gradient (urea and formamide). Electrophoresis was run for 12 h at 100 V. Banding profiles were visualized by staining the gel 1:10.000 (v/v) with SYBR Gold (Molecular Probes, Invitrogen Co., USA) for 30 min, followed by image capture using GelDoc-It^TS2^ Imager (UVP, Upland, CA, USA). DGGE banding profile clustering, using a dendrogram, was also carried out by using Phoretix 1D analysis software (TotalLab Ltd., UK). The correlation between bacterial communities (biological parameters) and chemical soil properties (ecological parameters) was visualized by non-metric multidimensional scaling (MDS) analysis using Primer 7 + Permanova software (Primer-E Ltd., Ivybridge, UK) (Clarke, 1993). The *in silico* analysis was also used to estimate the bacterial diversity by richness (S), Shannon–Wiener, and dominance by Simpson Index (D), represented by 1-D or 1-λ (Sagar and Sharma, 2012).

Ggt occurrence in soil samples: DNA extracts from soil samples were subjected to PCR using the primer set NS5 (5′-AAC TTA AAG GAA TTG ACG GAA G-3′) and GGT-RP (5′-TGC AAT GGC TTC GTG AA-3′) designed by Fouly and Wilkinson (2000) specifically for Ggt. The PCR conditions were as follow: an initial denaturation at 93°C for 3 min, followed for 93°C for 1 min, 52°C for 1 min, and 72°C for 1 min to 35 cycles, and finally with 72°C for 5 min. All soil samples were tested in triplicate, and pure *G. graminis* var *tritici* (Andrade et al., 2011) and *Aspergillus niger* DNA (code CCT-UFRO 15.62), obtained from La Frontera University Type Strain Culture Collection (http://ccct.ufro.cl/), were used as positive and negative controls, respectively. The presence of Ggt in soil samples showing positive Ggt reaction was also confirmed by the presence of take-all disease symptoms, chlorosis, and blackening roots in wheat seedlings in the pot assays.

### Putative Ggt-suppressive soil screening

Putative Ggt-suppressive soil screening was performed by two *in vitro* inhibition tests using rhizosphere soil samples as follows:

#### *In vitro* inhibition test on solid media

A first screening was carried out in order to evaluate rhizosphere soil capability of inhibiting Ggt growth on agar plates (Supplementary Figure 1A). Briefly, the Ggt inoculum was produced by growing the fungus on potato dextrose agar (PDA) medium at 25°C for 1 week. Agar disks (4-mm diameter) containing Ggt were aseptically incised and transferred to the center of agar plates containing fresh Luria Bertani (LB) and PDA medium (proportion 1:1). A hole of 10 mm was performed in the agar medium at a distance of 3 cm from Ggt inoculum, and 0.05 g of rhizosphere soil were placed in the agar hole. Ggt mycelia growth was registered at 3, 5, and 7 days of incubation at 25°C in the darkness, as described by Liu et al. (2011). A fraction of all soil samples was sterilized and samples included in the agar as negative controls. All tests were carried out in triplicate.

#### *In vitro* inhibition test in aqueous soil extracts

Because some soils contained elevated loads of microorganisms affecting fungal measurements on agar medium (categorized as un-determined in the *in vitro* test described in Section *In vitro* Inhibition Test on Solid Media), a second assay was performed in tubes with rhizosphere soil extracts (Supplementary Figure 1B). Briefly, 1 g of rhizosphere soil sample was suspended in 9 ml of sterile phosphate buffer saline (PBS; pH 7.4), and sonicated at 60% amplitude for 30 s. Then, 1 mL of supernatant was added to an eppendorf, and then inoculated with 1% Ggt inoculum. Soil extract tubes were incubated at room temperature for 3, 5, and 7 days, and fungal growth was estimated by quantification of fungal biomass by crystal violet (CV) staining as follows. After incubation, soil extract samples were washed with distilled water and fixed with 500 μl methanol for 15 min at room temperature. Later, they were centrifuged at 13,000 rpm × 1 min, supernatants were discarded, and tubes were air-dried; 400 μl of CV was added to each tube and incubated for 5 min. Tubes were washed three times with distilled water. Finally, 400 μl of acetic acid (33% v/v) were added and kept in the tubes for 5 min. The absorbance of the obtained solution was determined in triplicate in a multi-plate reader at 590 nm (Silva et al., 2009, Supplementary Figure 1B).

Nine putative suppressive soils from the inhibition tests, in both agar plates and soil extract tubes, were used for the greenhouse assay, using soil 1 as Ggt-conducivepositive control.

### Take-all disease suppression assay in greenhouse

#### Inoculum preparation

The characterization of the pathogen as Ggt was done based on the sequencing of ribosomal internal transcribed spacer 2 region (ITS2). ITS2 was amplified by touchdown polymerase chain reaction (PCR) with primer sets fITS9 (5′-GAACGCAGCRAAIIGYG-3′) and ITS4 (5-′TCCTCCGCTTATTGATATGC-3′), using the following conditions: an initial denaturation at 95°C for 3 min, followed by 25 cycles—each at 95°C for 30 s, with an annealing step with a 0.5°C decrease—each cycle from 65°C to 52.5°C, and extension at 72°C for 30 s. Twenty-five additional cycles were then carried out at 95°C denaturation for 30 sec, 55°C annealing, primer extension at 72°C for 30 s, and a final extension step of 7 min at 72°C. The PCR products were purified and sequenced by Austral-Omics (Universidad Austral of Valdivia-Chile). The sequence was compared with those in the GenBank database, fungal identity was confirmed (99%), and then deposited under accession no. KY689233.

The Ggt inoculum was prepared as follows: oat kernels were soaked in water for 24 h and sterilized for 3 consecutive days. Then, Ggt pathogenic isolate discs were grown on PDA for 7 days, put on the sterile oat, and maintained at room temperature for 20 days. Colonized oat kernels were blended, sieved to a particle size of 0.5–1.0 mm, and stored at 5°C until usage (Andrade et al., 2011).

#### Greenhouse assay

Nine putative suppressive soils (number 2, 3, 4, 6, 11, 13, 14, 15, and 16), selected according to results obtained in the *in vitro* tests (Section Putative Ggt-Suppressive Soil Screening), were tested in their ability to suppress take-all disease *in planta* under greenhouse conditions. Plastic containers enclosing 200 g of soil were used in quintuplicate. Wheat seeds Otto cv were surface sterilized (15% ethanol plus 1% sodium hypochlorite for 2 min) and 5 seeds were used for each treatment. Plants were watered every 3 days, and Taylor and Foyd nutrient solution (Taylor and Foyd, 1985) was applied each 15 days.

The experimental design consisted in sterile (heat-treated) rhizosphere soil to determine disease level incidence, discarding, or diminishing the effect of soil microorganisms; untreated, air-dried soil (natural) to determine the effect of soil native microorganisms; and, sterile soil +1% untreated, natural soil (ster+1% nat) to assess suppression transferability to sterile soils. Suppression transferability has been shown to occur when natural soils are added in as low as 1% (v:v) to non-suppressive soils, as reported earlier (Shipton et al., 1973; Andrade et al., 1994). Ggt inocula were applied at 0.1% in relation to soil weight (2 g), and all treatments were also performed in soil without Ggt inocula as controls. After 40 days, plants were carefully removed from the soil, weighted and the root blackening percentage was determined.

#### Ggt presence in plant tissues

The Ggt presence was evaluated in roots of PCR infected and non-infected plants. Roots were individually assessed for infection and root blackening percentage was evaluated against a white background. Shoots were carefully separated from the roots, placed into individual paper envelopes, and dried at 70°C for 72 h, to obtain shoot dry weight. In order to confirm Ggt infection, total DNA from wheat infected tissues was extracted with soil DNA Isolation Kit (Ultraclean, Mo-Bio Laboratories) according to manufacturer's instructions. Specific Ggt DNA fragments were amplified by using NS5 and GGT-RP primer sets, as described in Section Bacterial Community Composition and Ggt Detection in Soil Samples. Pure DNA extracts from Ggt and *A. niger* collection strains were used as positive and negative control, respectively.

#### Presence of 2,4-diacetylphloroglucinol-producing bacteria

The presence of 2,4-DAPG-producing bacteria was also evaluated by PCR. Total DNA from rhizosphere soil was extracted with soil DNA Isolation Kit (Ultraclean, Mo-Bio Laboratories) according to manufacturer's instructions. Specific primer sets B2BF (5′ACC CAC CGC AGC ATC GTT TAT GAG C-3′) and BPR4 (5′CCG CCG GTA TGG AAG ATG AAA AAG TC-3′), which target the *phlD* gene (encoding a polyketide synthase that synthesizes monoacetylphloroglucinol, the precursor to 2,4-DAPG that is essential for the phloroglucinol biosynthesis) were used in the PCR reaction (Gardener et al., 2001). PCR conditions were: an initial denaturation at 95°C for 3 min, followed by 35 cycles each at 95°C denaturation for 1 min, 60° annealing for 1 min, 72°C extension for 1 min, and final extension step for 10′ at 72°C. Pure DNA extracts from *Pseudomonas* spp. (SA 32A) and *Enterobacter* spp. (RJAL6) strains were used as positive and negative controls, respectively (Mora et al., 2017).

#### Bacterial community structure in suppressive soils

The bacterial community composition in the rhizosphere suppressive soils from the greenhouse assay was examined by DGGE as described in Section Bacterial Community Composition and Ggt Detection in Soil Samples. The similarity between bacterial communities was visualized by non-metric multidimensional scaling analysis (MDS), using Primer 7 software (Primer-E Ltd., Ivybridge, UK), which showed a Bray–Curtis similarity index higher than 50% and 0.14 stress values (Clarke, 1993).

### Statistical analyses

Data normality was analyzed according to Kolmogorov's test. Data obtained in Section *In vitro* Inhibition Test on Solid Media (*in vitro* plate assay) were analyzed by a one-way analysis of variance (ANOVA) and compared by Tukey test, using SPSS software (SPSS, Inc.). Comparisons between inoculated and non-inoculated samples from screening 2 were made, and Student *t*-test was used for related samples with 95% confidence interval. For the greenhouse assay multivariate analysis of variance (MANOVA) and comparisons were carried out for each set with Tukey's test by SPSS software (SPSS, Inc.). Values were given as means ± standard errors. Differences were considered significant when the *P* value was lower than or equal to 0.01. The microbial diversity analysis was described above.

## Results

### Collected soils grouped according to their chemical composition

In order to determine the chemical composition of the collected soils, chemical analyses were performed using triplicate samples of each soil. The main chemical parameters that were measured are shown in Table 2. In general, soil samples showed values of available P from 5.6 (soil 13) to 60 mg kg^−1^ (Soils 1 and 4). The pH ranged from 5.0 (soils 11 and 12) to 6.4 (soil 15). The OM contents varied from 6% (soil 14) to 15% (soils 6, 7, and 15). The higher values of S bases and Al saturation were observed in soil 15 (29.4 cmol_(+)_ kg^−1^) and soil 11 (14%), respectively; whereas lower values were observed in soil 6 (4.7 cmol_(+)_kg^−1^) and soil 15 (0 cmol_(+)_kg^−1^). In addition, the PCA analysis showed that soils were grouped based on their chemical composition and soil classification, Andisol and Ultisol (Figure 1B). Several soil groups were formed, soils collected from Perquenco, Las Cardas, Momberg, Quilaco, Membrillar, and Lufquentue clustered together (soils 1, 2, 3, 4, 5, 7, 9, 10, and 16, respectively), soils from Quilaco (6) and Boyeco (11 and 12), and three soils collected from Lufquentue (soils 13, 14, and 15) and Quilaco (soil 8) did not cluster with any other group.

**Table 2:** Average values for some chemical properties of rhizosphere soils used in this study.

**Soil**	**P (mg kg^−1^)**	**K (cmol + kg^−1^)**	**pH (H_2_O)**	**OM (%)**	**Al sat^†^ (%)**	**CICE (cmol + kg^−1^)**	**Σ basis (cmol _(+)_ kg^−1^)**
1	60.3 ± 0.8^a^^*^	2.8 ± 0.1^bc^	5.9 ± 0.1^b^	12.2 ± 0.1^d^	0.6 ± 0.0^hij^	16.9 ± 0.0^e^	16.9 ± 0.0^e^
2	41.5 ± 0.9^cd^	1.2 ± 0.0^f^	5.3 ± 0.0^f^	10.9 ± 0.2^e^	9.9 ± 0.1^c^	11.0 ± 0.1^h^	9.9 ± 0.1^i^
3	22.2 ± 0.6^h^	3.0 ± 0.1^b^	5.8 ± 0.0^bc^	10.4 ± 0.3^ef^	0.9 ± 0.1^fgh^	18.5 ± 0.1^d^	18.3 ± 0.1^d^
4	59.8 ± 0.2^a^	0.6 ± 0.0^hi^	5.7 ± 0.0^bc^	15.1 ± 0.1^ab^	1.1 ± 0.0^fg^	9.4 ± 0.0^i^	9.3 ± 0.0^i^
5	40.1 ± 1.0^d^	2.6 ± 0.1^c^	5.7 ± 0.1^bc^	15.3 ± 0.3^a^	0.7 ± 0.0^fgh^	13.9 ± 0.0^g^	13.8 ± 0.0^g^
6	22.2 ± 0.1^h^	0.4 ± 0.0^ij^	5.4 ± 0.1^def^	15.9 ± 0.2^a^	3.3 ± 0.1^d^	4.8 ± 0.1^jk^	4.7 ± 0.1^j^
7	33.1 ± 0.1^f^	1.2 ± 0.0^f^	5.7 ± 0.1^bc^	15.4 ± 0.2^a^	0.6 ± 0.1^ghi^	10.9 ± 0.1^h^	10.8 ± 0.1^h^
8	53.4 ± 0.1^b^	9.2 ± 0.1^a^	6.3 ± 0.0^a^	13.9 ± 0.2^c^	0.0 ± 0.0^j^	28.1 ± 0.2^b^	28.1 ± 0.2^b^
9	37.5 ± 0.2^e^	2.2 ± 0.1^d^	5.6 ± 0.0^cde^	10.9 ± 0.3^e^	2.3 ± 0.0^e^	11.2 ± 0.2^h^	11.0 ± 0.2^h^
10	42.7 ± 0.2^c^	2.1 ± 0.1^de^	5.6 ± 0.1^cde^	14.0 ± 0.3^bc^	1.2 ± 0.0^fg^	9.9 ± 0.1^i^	9.8 ± 0.1^i^
11	16.1 ± 0.3^i^	0.8 ± 0.0^gh^	5.0 ± 0.0^g^	7.7 ± 0.1^g^	14.0 ± 0.4^a^	5.8 ± 0.1^j^	5.0 ± 0.0^j^
12	13.5 ± 0.2^j^	0.2 ± 0.0^j^	5.0 ± 0.0^g^	9.6 ± 0.1^f^	12.5 ± 0.1^b^	6.3 ± 0.0^j^	5.5 ± 0.0^j^
13	5.6 ± 0.4^k^	0.9 ± 0.0^gh^	5.6 ± 0.0^cd^	13.5 ± 0.2^c^	0.5 ± 0.0^hij^	15.7 ± 0.1^f^	15.6 ± 0.1^f^
14	30.0 ± 0.3^g^	1.1 ± 0.0^fg^	6.2 ± 0.1^a^	6.1 ± 0.3^h^	0.1 ± 0.0^ij^	24.8 ± 0.4^c^	24.7 ± 0.4^c^
15	16.0 ± 0.3^i^	0.3 ± 0.0^j^	6.4 ± 0.1^a^	15.2 ± 0.2^a^	0.0 ± 0.0^j^	29.4 ± 0.3^a^	29.4 ± 0.3^a^
16	54.2 ± 0.1^b^	1.8 ± 0.0^e^	5.3 ± 0.1^ef^	7.9 ± 0.3^g^	0.6 ± 0.0^ghi^	11.6 ± 0.1^h^	11.5 ± 0.1^h^

† *Calculated as Al/cation exchange capacity [Σ (K, Ca, Mg, Na, and Al)] × 100, n = 3*.

* *Different letters in same column denote significant differences (Tukey's test, P ≤ 0.05)*.

### Bacterial community composition is related to chemical soil composition and differs in bulk and rhizosphere soils in terms of dominance and diversity

**Figure 2:**
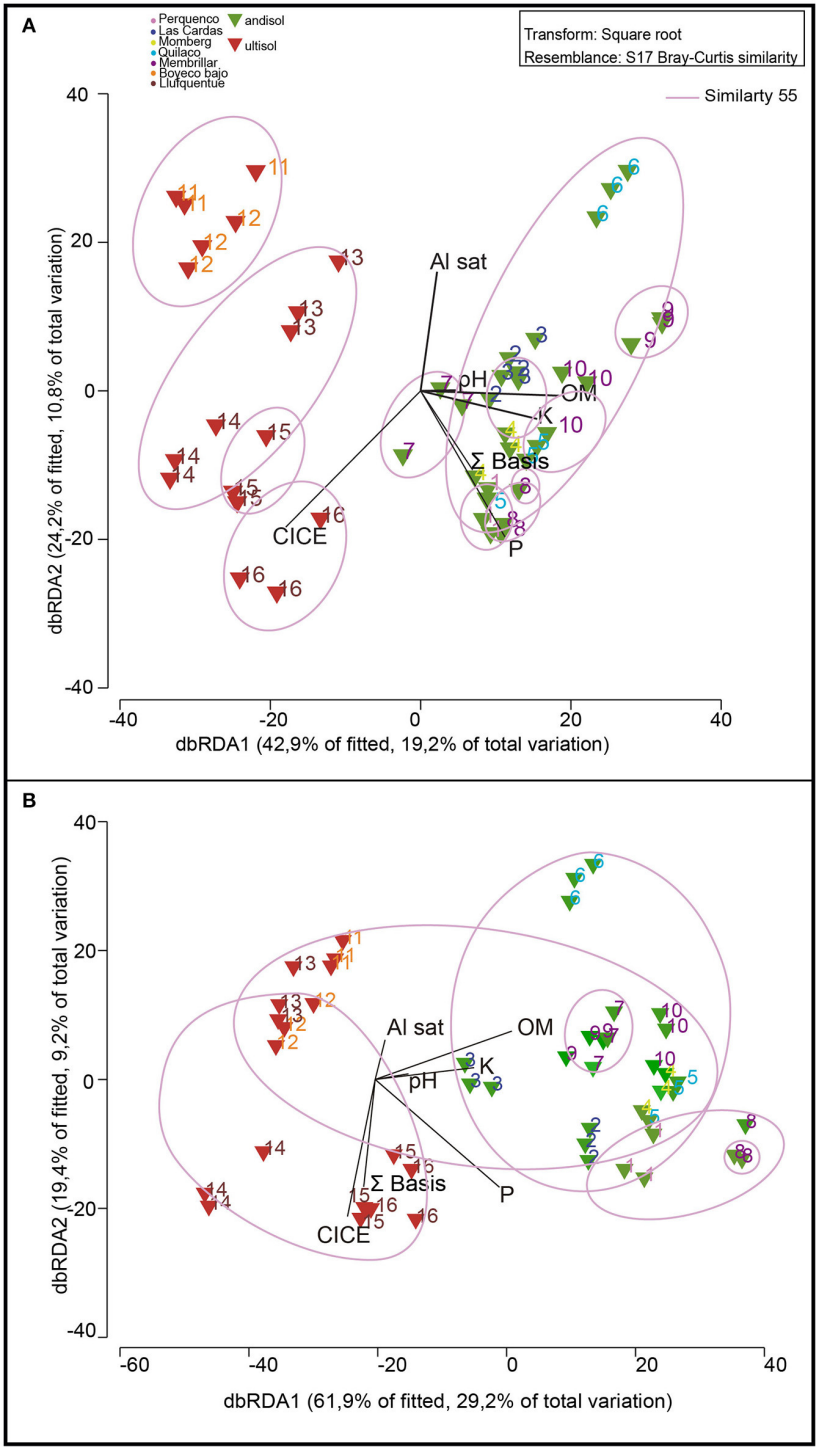
Non-metric Multidimensional Scaling (NMDS) analysis of the 16 soils used in this study based in DGGE profiles of bacterial communities in relation with soil parameters (P, K, OM, Al sat, CICE, and Σ basis). Color of numbers represent sector of sampling and triangle colors indicate type of soil: andisol (green) and ultisol (red). The length and position of the black lines (soil parameters) indicate correlation strength and direction of significant variables (*P* < 0.05) with the microbial community of rhizosphere **(A)** and bulk soil **(B)**.

Rhizosphere community composition is highly related to soil classification as revealed by MDS, in which microbiology and soil chemical/environmental properties were analyzed (Figure 2A). Samples from Andisol and Ultisol were notably different from each other, with 55% similarity. According to the Spearman correlation, Ultisol soils were more significantly related with chemical parameters than Andisol soils (*r*^2^ = 0.90 and *r*^2^ = 0.59, respectively, Table 3). Bulk soils were also separated according to the chemical soil composition, but this difference was not significant, and all samples were grouped at 55% (Figure 2B). In this sense, both Andisol and Ultisol were equally related with soil chemical parameters (*r*^2^ = 0.83, Table 3).

**Table 3:** *R*^2^ values and significance level for NMDS ordination and environmental variables.

	**Rhizosphere soil**			**Bulk soil**		
**Soil parameters**	**All samples**	**Andisol**	**Ultisol**	**All samples**	**Andisol**	**Ultisol**
P (mg kg^−1^)	0.001^**^	0.020^*^	0.001^**^	0.001^**^	0.003^**^	0.005^**^
pH (H_2_O)	0.027^**^	0.037^*^	0.05^*^	0.257	0.513	0.508
K (mg kg^−1^)	0.028^**^	0.09	0.001^**^	0.128	0.001^**^	0.001^**^
OM (%)	0.003^**^	0.003^**^	0.001^**^	0.001^**^	0.014^*^	0.016^*^
Al saturation (%)	0.008^**^	0.006^**^	0.001^**^	0.167	0.066	0.08
CICE (cmol+ kg^−1^)	0.003^**^	0.001^**^	0.507	0.007	0.968	0.97
Σ basis (cmol_(+)_kg^−1^)	0.001^**^	0.012^*^	0.123	0.069	0.036^*^	0.023^*^
*R*^2^ (Spearman)	0.45	0.59	0.90	0.47	0.83	0.83

* *Represents statistically significant correlation (P < 0.05)*,

** *represent statistically significant correlation (P < 0.01)*.

**Figure 3:**
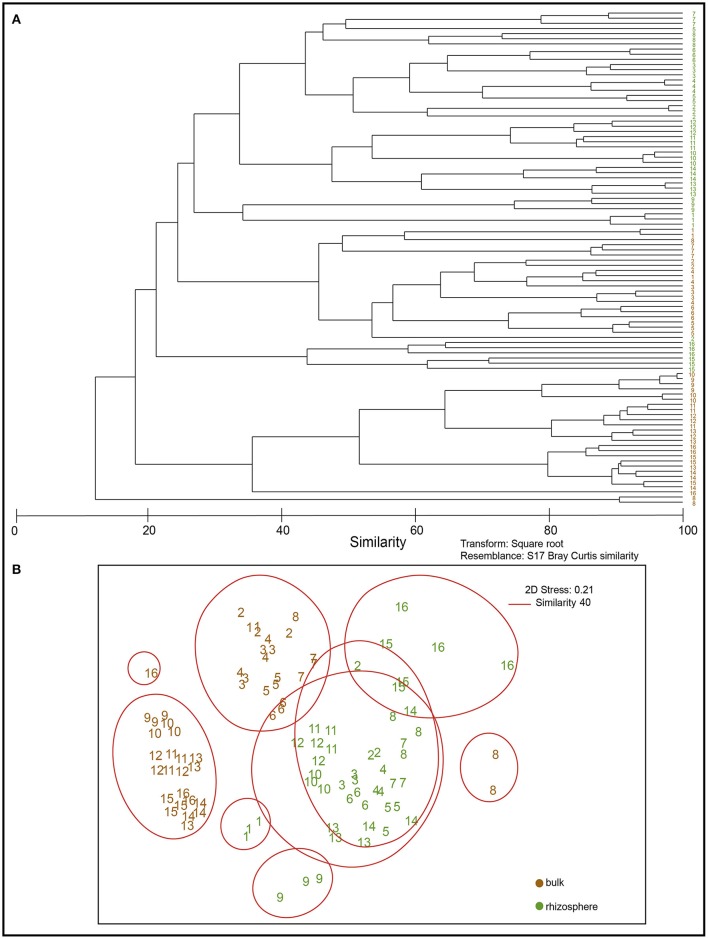
Dendogram of DGGE profiles **(A)** and non-metric Multidimensional Scaling (NMDS) based on denaturing gradient gel electrophoresis (DGGE) profiles of bacterial communities present in bulk (brown) and rhizosphere (green) soil samples **(B)**.

Differences in bacterial community structures between bulk and rhizosphere soil were revealed by MDS analysis, based on DGGE banding profiles. In relation to bulk soils, the nMDS analysis revealed the existence of major groups at 40% similarity formed by rhizosphere soils indistinctly grouped, all correlated among them. In general, bulk soils were separated into two main groups formed mainly by Andisol (soil 1, 2, 3, 4, 5, 6, 7) and Ultisol soils (11, 12, 13, 14, 15, 16) (Figure 3).

Regarding the microbial diversity in bulk and rhizosphere soils (Supplementary Figure 2), in general, the Shannon index (H') showed values <2.0 for bulk (except soils 5 and 6 from Quilaco) whereas it reached values ~2.5 for rhizosphere soil, indicating a lower diversity in bulk than in rhizosphere soils. A similar trend was observed in the case of species number (S). The lowest diversities (<1.0) and species richness (S) were observed in bulk soils from Membrillar (soils 9 and 10) whereas highest diversity values were obtained in rhizosphere soils from Quilaco (soil 5 and 6). As for bulk soils, rhizosphere soils taken from Membrillar also showed lower diversity values (≤2.0). In contrast, less dominance values in rhizosphere soils compared with bulk soils were also observed by Simpsons (D) index represented by 1-D, in which the lower values indicate major dominance of species. Thus, samples 9 and 10, which showed less diversity according to the Shannon index, also showed major dominance of species. Therefore, bulk soils showed lower biodiversity and major dominance compared to rhizosphere soils (Supplementary Figure 2), and we observed a direct Pearson correlation between both indexes (*P* < 0.01, data not shown).

### Screening for putative Ggt-suppressive soils

**Figure 4:**
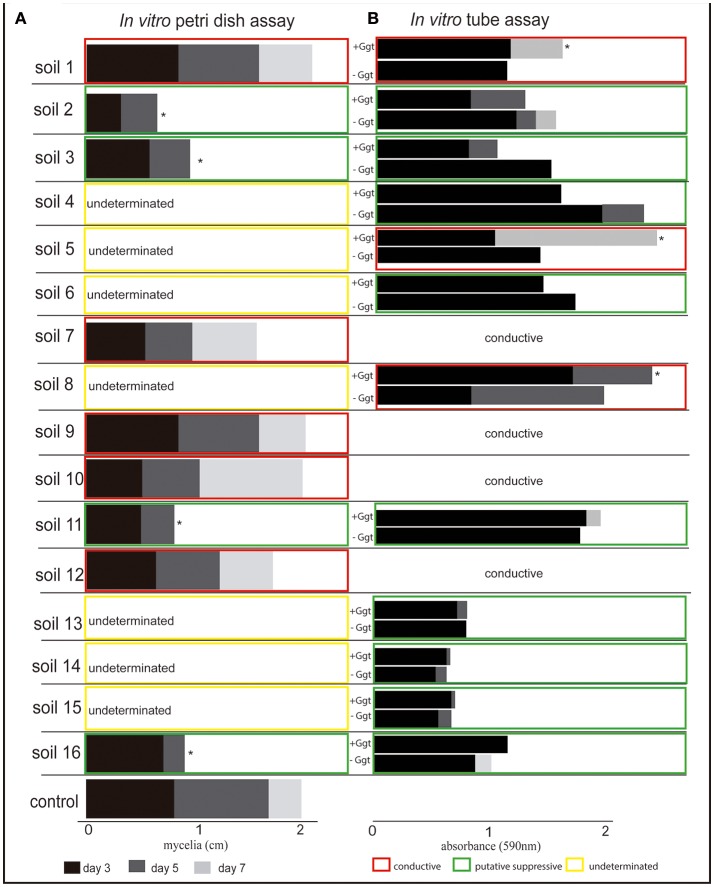
*In vitro* assays to determine the inhibition of *Gaeumannomyces graminis* growth in the presence of rhizosphere soil samples. **(A)** Shows Ggt mycelial growth on solid media supplemented with the different rhizosphere soils after 3, 5, and 7 days of incubation. **(B)** Shows Ggt fungal biomass in liquid rhizosphere soil extracts after 3, 5, and 7 days of incubation. Asterisks indicate significant differences (Tukey's test, *p* ≤0.05) in relation to the positive control (solid media assay) and negative control (liquid assay).

According to the *in vitro* inhibition test on solid media (Figure 4A and Supplementary Figure 1A), four soils from Las Cardas (soils 2 and 3), Boyeco (soil 11), and Llufquentue (soil 16) were considered as suppressive against Ggt, since Ggt growth was significantly inhibited when compared with the positive control. However, several soils could not be properly evaluated based on this assay and were classified as undetermined. A second evaluation, using the *in vitro* inhibition test in aqueous soil extracts (Figure 4B and Supplementary Figure 1B) suggested the presence of nine Ggt-suppressive soils. They were collected from Las Cardas, Momberg, Quilaco, Boyeco, and Llufquentue (soils 2, 3, 4, 6, 11, 13, 14, 15, and 16, respectively). In line with the assay on solid agar, this test also showed that soils collected from Membrillar (soils 7, 9, 10, and 12) were Ggt conducive. This assay also revealed the presence of other Ggt-conducive soils (soils 5 and 8), including the positive control soil 1. In summary, based on *in vitro* experiments, 9 soils were determined as suppressive, and 7 as conducive (Figure 4B).

### Suppressive soil greenhouse assay

#### Incidence of take-all disease

**Figure 5:**
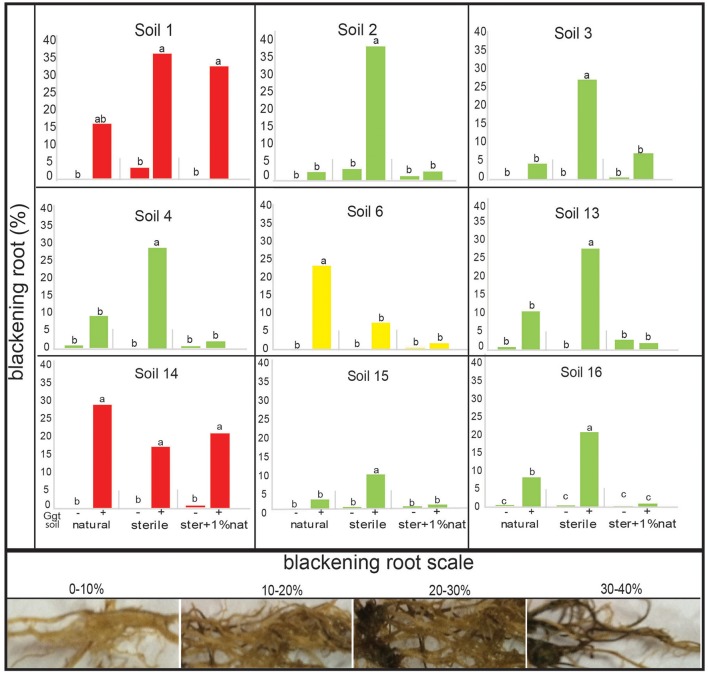
Take all disease symptoms (root blackening) in wheat plants from the suppressive soil bioassay in greenhouse. Treatments were: natural soil (natural), sterile soil (sterile), and sterile soil supplemented with 1% of natural soil (ster+1% nat), inoculated (+) or not (−) by Ggt (*n* = 5). The disease index scale used is represented by the pictures in the bottom panel. Tukey's test was used to compare treatments means, values followed by the same letter do not differ at *P* ≤ 0.05 (*n* = 5). Green bars represent suppressive soils, red bars conducive soils, and yellow bars represent undeterminated soil.

Firstly, we aimed to establish a positive control for take all decline and soil conduciveness for our suppressive soil assays. Plants growing in soil 1 showed clear disease symptoms including leaf chlorosis and blackening root, distinctive take-all disease symptoms. We confirmed the presence and the identity of Ggt in the rhizospheric soil, extracting the total DNA and using Ggt specific primers. The PCR amplification confirmed that soil 1 was positive in terms of Ggt presence (Supplementary Figure 3). Then, to confirm the ability to suppress take-all disease in the putative nine rhizosphere soils selected through the *in vitro* assays (Figure 4), an assay with the pathosystem wheat-Ggt was performed under greenhouse conditions by growing wheat in Ggt-inoculated and non-inoculated soils. Take-all disease symptoms were evident in all inoculated soils, except in soil 11 (data not shown). The origin of the symptoms was confirmed by Ggt DNA amplification with Ggt specific primers of infected plants; the amplification band was present in all Ggt-inoculated rootswhereas it was absent in the non-inoculated plants (Supplementary Figure 4). In order to determine whether Ggt suppressiveness was due to microbial community or to soil physico-chemical characteristics, sterilization by heat treatment of the soils was performed. The plants grown in the greenhouse on heat-treated sterilized soils showed higher disease symptoms than in the corresponding natural soils (Figure 5): the percentage of blackening root for plants growing in sterile soils ranged between 10 and 40%, while only between 3 and 10% for natural soils, except for soils 1, 6, and 14 (Figure 5). In fact, no significant differences were found between sterile and natural soil in the case of soils 1 and 14 (blackening roots levels ranging from 15 to 35%).

**Figure 6:**
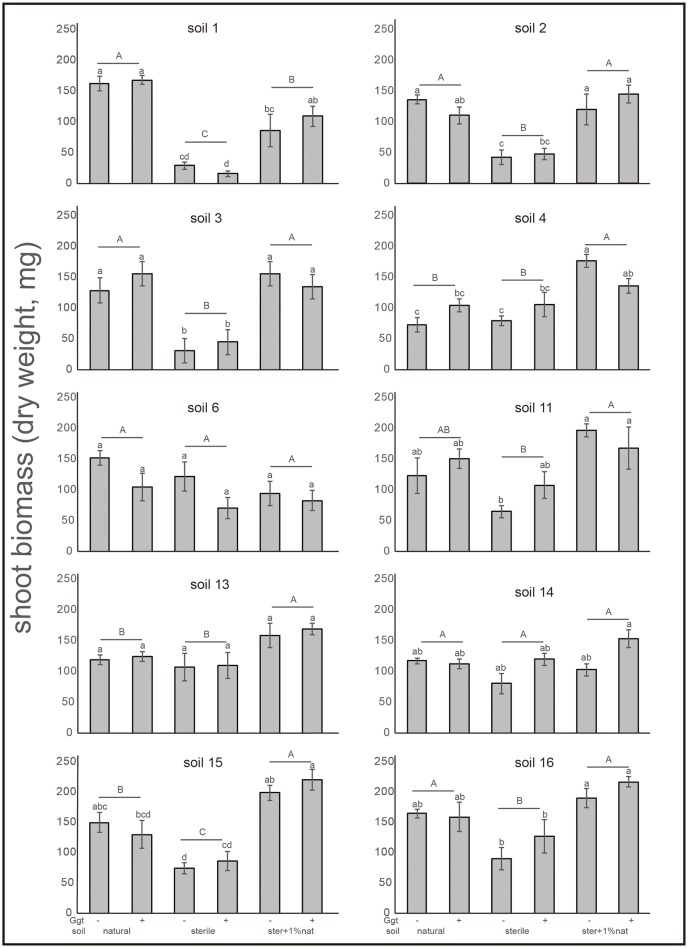
Shoot biomass (dry weight) of wheat plants from suppressive soil assay in greenhouse. Treatments were: natural soil (natural), sterile soil (sterile), and sterile soil supplemented with 1% of natural soil (ster+1% nat)- and inoculated (+) or not (−) by Ggt (*n* = 5). Tukey's test was used to compare treatments means, values followed by the same letter do not differ at *P* ≤ 0.05 (*n* = 5).

A negative correlation between blackening roots and biomass was also observed. Thus, the lower biomass was found in the sterile Ggt-inoculated soil treatments, where plants showed higher plant infection, with the exception, again, of soils 6 and 14, in which plant biomass was similar in sterile and natural soils (Figure 6). In treatments where a 1% of the natural soils was added to the sterile soils, biomass production increased significantly (*P* ≤ 0.05). In fact, in soils 4, 13, and 15 the highest biomass for this treatment was found, being similar in the rest of the soils. Only in soil 1 (Ggt-conducive positive control) plant biomass was lower in the 1% natural soil supplemented treatment.

#### Presence of 2,4-DAPG producing bacteria

2,4-DAPG-producing bacteria have been reported as major take-all disease suppressors in soils (Kwak et al., 2012). Accordingly, we checked for their presence in our selected suppressive soils. However, we found that only soil 15 was positive for the phlD gene, essential in the DAPG biosynthetic pathway (Gardener et al., 2001). The rest of suppressive soil samples did not show the presence of amplicons for this gene (Supplementary Figure 5), suggesting that other metabolic pathways should be responsible for suppressiveness in those soils.

#### Rhizosphere bacterial community composition in suppressive and conducive soils

**Figure 7:**
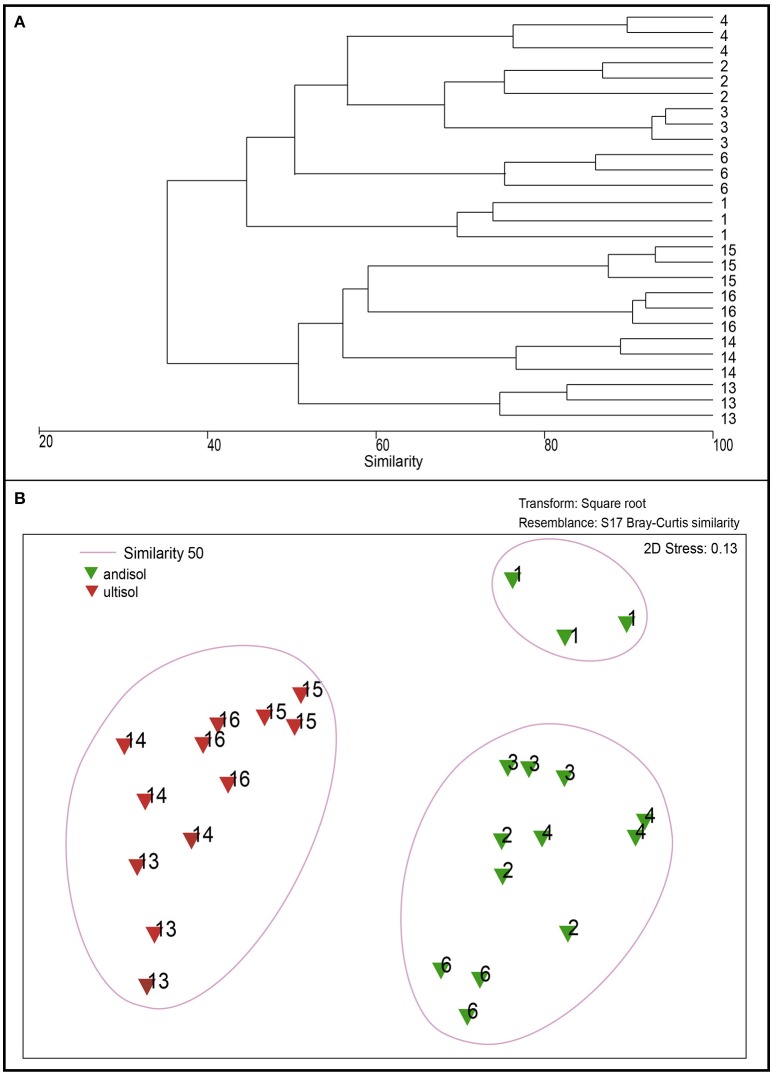
Dendrogram of DGGE profiles **(A)** and non-metric Multidimensional Scaling (NMDS) analysis **(B)** of bacterial communities in the rhizosphere of soils from the greenhouse experiment (soils 2, 3, 4, 13, 14, 15, and 16) compared with Ggt-conducive soil (soil 1).

Regarding the composition of the rhizosphere bacterial communities (as revealed by DGGE analysis), the dendrogram showed differences between suppressive and conducive soils in the case ofAndisols (Figure 7). These results were confirmed by using MDS analysis, showing clear differences between the conducive control soil (soil 1) and the rest of treatments in Andisol (55% similarity). However, in the case of Ultisols in which the bacterial communities were more significantly related with the soil chemical parameters than in Andisol soils (Table 3), this tendency was not observed (Figure 7B).

## Discussion

Several studies have described the importance of soil microorganisms as suppressive agents against phytopathogens worldwide, including soils with chemical properties similar to Chilean agricultural soils, such as, New Zealand soils (Bithell et al., 2013; Chng et al., 2013, 2015; Perez et al., 2016). However, despite of the great potential that Chilean suppressive soils offer in terms of microbial diversity and potential for the development of biocontrol strategies, their studies are extremely limited (Andrade et al., 2011). This topic acquires special relevance when ancestral extensive agriculture has been applied for a long term by native communities; Mapuche people cultivate in small areas to produce their own agricultural products using rustic metal tools and low inputs (Montalba-Navarro, 2004). This ancestral production could be replaced by more aggressive and intensive modern agriculture techniques, with the possible subsequent loss of diversity and suppressive potential of soil microbial communities. Under this scenario, it is essential to identify suppressive soils in these areas. In this work we screened the ability of different agricultural soils from southern Chile to suppress take-all disease in wheat plants. Suppressive soils were collected from little farms mainly belonging to indigenous Mapuche communities from Southern Chile, who practice monoculture and cultivate small subsistence wheat areas.

### Soil chemical parameters and their relation with microbial community composition

Our results on soil chemical properties showed that 25% of the studied soils presented a low content of available P (< 20 mg kg^−1^), moderate acidity (pH < 5.5), and high Al saturation (>10%), which are main characteristics of agricultural soils from southern Chile (Mora et al., 2006). In general, Andisols grouped together when considering the chemical parameters, whereas Ultisols were more diverse. However, when we compared microbial diversity we observed a direct correlation with soil chemistry mainly in rhizospheric soils, and more significantly for Ultisols. Similar results were found by Smalla et al. (2007) despite the amplified fragments comprised different variable regions and lengths, DGGE, T-RFLP, and SSCP analyses led to clustering of fingerprints, which correlated with soil physico-chemical properties. In Chilean Andisol soils, Jorquera et al. (2014) also showed that soil chemistry influenced the composition of rhizobacterial communities, and in Ultisol soils from China, Li et al. (2017) found through nMDS analyses that microbial communities also correlated with soil chemical parameters and fertilization strategies. Although there is some similarity in microbial composition between rhizosphere and bulk microbial composition (de Ridder-Duine et al., 2005), in our study rhizosphere soils were more diverse in terms of richness and dominance than bulk soils. In fact, it is known that the amount of microorganism around the rhizosphere is 10- to 1,000-fold higher than that found in bulk soil due to rhizodeposition (Doughari, 2015; Glick, 2015).

### Suppressive soil identification in southern chile

In terms of potential take-all disease suppression, *in vitro* Ggt-growth inhibition tests with 16 different soils allowed to identify 9 potentially suppressive soils, and 6 of them were later confirmed to be suppressive in plant bioassays under greenhouse conditions (soil 2, 3, 4, 13, 15, and 16). In general, these soils were cultivated with wheat monoculture and natural pasture for more than 10 years, a management similar to that described previously for other suppressive soils in South Chile (Andrade et al., 2011). Regarding the timing, take-all suppression appeared after 4–6 years of wheat monoculture (McSpadden Gardener and Weller, 2001), and even later, although they can also occur in soils with 3–4 years of monoculture under relatively high pathogen concentrations (Chng et al., 2015). In fact, early studies by Baker and Cook (1974) showed that 3 years of successive wheat cropping could be sufficient for the development of specific suppression. As an exception, soil 2, withrotation based in wheat-triticale and oat for more than 10 years, was also found to be Ggt-suppressive. It is well known that oat roots produce saponin avenacin, a glycosylated triterpenoid secondary metabolite with antifungal properties that has been involved in determining oat resistance to soil fungal pathogens (Osbourn et al., 1994; Freeman and Ward, 2004). On the other hand, wheat plants grown in soil 11, characterized by a very high humidity, showed no symptoms of Ggt infection in roots. This is in agreement with earlier reports by Nish (1973), who studied Ggt survival in the field under controlled conditions, and showed a significant reduction in Ggt incidence in wet cool soils.

In greenhouse assays, wheat plants grown on sterile Ggt-inoculated soils presented the highest disease incidence and the lowest biomass production, suggesting the relevance of the autochthonous soil microbial communities on plant growth promotion and disease suppression. The effect observed in sterile soils—increased susceptibility and reduced biomass—was improved when they were supplemented with 1% of the same natural soils, except in the case of the conducive soils 1 and 14. This improvement also confirms the role of the microbial communities originally present in the take-all disease suppressive soils and Ggt control. This characteristic is known as specific suppression (Cook, 2003; Andrade et al., 2011; Chng et al., 2015). It is noteworthy that we did not find any relation between suppressive soils and the presence of 2,4-DAPG-producing bacteria since its presence was only detected in one out of the six confirmed suppressive soils. The low occurrence of 2,4-DAPG determined by *phlD* gene, suggests that other mechanism(s) or antifungal compound(s) are synthetized by native soil microorganisms that could contribute to the effective biocontrol against Ggt. Thus, strains related to soil suppressiveness seem to be differentially shaped by multiple soil factors (Imperiali et al., 2017). Therefore, further studies are required to identify the mechanisms involved in Ggt disease suppression.

### Suppressive soils and microbial community composition

As mentioned above, the selected suppressive soils did not group together in relation to their chemical properties and geographical origin. However, when bacterial communities were analyzed by DGGE, within Andisols, suppressive soils grouped separately with respect to the control conducive soil 1, but not for Ultisols, in which suppressive soils grouped together with soil 14, also classified as conducive. This could be attributed to the high relation between Ultisol soils and soil chemical parameters when compared to that relation in Andisol soils (*r*^2^ = 0.90 and 0.59, respectively). Future research should explore which specific microbial groups act directly upon Ggt suppression and how rhizosphere microbial communities are selected and regulated by the plant rhizosphere, especially in the presence of the phytopathogens. Moreover, identifying the bacterial groups and their antagonist mechanisms, as well as exploring the potential stimulation of plant defense mechanisms are pivotal for the development of biocontrol strategies based on the use of suppressive soils. With this aim, future research should tackle the multifactor soil-microbiome-plant-pathogen systems, considering not only direct antifungal activities in the rhizosphere, but also potential stimulation of plant defense and microbe-selection mechanisms.

## Conclusions

Suppressive soils represent an important microbial source for the biocontrol of soil-borne pathogens, and their identification and characterization is crucial since many of these soils may be lost by the increase of intensive agriculture practices worldwide. Here, we identified six suppressive soils against take-all disease, which have been managed under ancestral and rudimental agronomic practices by Chilean indigenous Mapuche communities. Then, we showed that suppressive activity in the tested soils correlated with the microbial community composition and not with the chemical properties and geographical origin of the studied soils. The key role of the soil microbial communities in Ggt suppression was confirmed in assays with sterile (heat-treated) suppressive soils where Ggt suppresiveness was completely lost, and recovered again through the addition of 1% of the corresponding non sterile natural suppressive soil. Our understanding of microbial communities in suppressive soils as well as the mechanisms acting in disease suppression in the rhizosphere must be considered as a valuable tool for the development of sustainable control of soil-borne pathogen (such as, take-all disease) in agriculture.

## Author contributions

PD, SV, MJ, MM designed the research. MP and VC supervised the study. PD organized the soil sampling and chemical soil analysis. SV performed the greenhouse experiments. VC analyzed the data. PD and MP wrote the manuscript and authors critically revised the manuscript and approved the final version.

### Conflict of interest statement

The authors declare that the research was conducted in the absence of any commercial or financial relationships that could be construed as a potential conflict of interest.

## References

[B1] Andrade O. Campillo R. Peyrelongue A. (2011). Soils suppressive against *Gaeumannomyces graminis* var. tritici identified under wheat crop monoculture in southern Chile. Cs. Invest. Agric. 38, 345–356. 10.4067/S0718-16202011000300004

[B2] Andrade O. Mathre D. E. Sands D. C. (1994). Suppression of *Gaeumannomyces graminis* var *tritici* in montana soils and its transferability between soils. Soil. Biol. Biochem. 26, 397–402. 10.1016/0038-0717(94)90289-5

[B3] Arismendi N. Doussoulin H. Moya E. (2012). Determinación de Pseudomonas productoras de 2,4 diacetilfloroglucinol en cultivos de trigo en el sur de Chile, in XXI Congreso Chileno de Fitopatología, 17-18-19 de octubre 2012 (Puerto Varas Chile).

[B4] Baker K. F. Cook R. J. (1974). Biological Control of Plant Pathogens. San Francisco, CA: Freeman.

[B5] Bertsch P. M. Bloom P. R. (1996). Aluminum, in Methods of Soil Analysis, Part 3—Chemical Methods, ed Bigham J. M. (Madison, WI: Soil Science Society of America), 526–527.

[B6] Bithell S. L. S. McKay A. Butler R. New T. Bag P. Zealand N. (2012). Predicting take-all severity in second-year wheat using soil DNA concentrations of *Gaeumannomyces graminis* var. tritici determined with qPCR. Plant Dis. 96, 443–451. 10.1094/PDIS-05-11-0445 30727140

[B7] Bithell S. L. Butler R. C. McKay A. C. Cromey M. G. (2013). Influences of crop sequence, rainfall and irrigation, on relationships between *Gaeumannomyces graminis* var. tritici and take-all in New Zealand wheat fields. Australas. Plant Pathol. 42, 205–217. 10.1007/s13313-012-0168-9

[B8] Bull C. T. Weller D. M. Thomashow L. S. (1991). Relationship between root colonization and suppression of *Gaeumannomyces graminis* var. *tritici* by *Pseudomonas fluorescens* strain 2-79. Phytopathology 81, 954–959. 10.1094/Phyto-81-954

[B9] Chng S. F. Stewart A. Cromey M. G. Dodd S. L. Butler R. C. Jaspers M. V. (2013). Effects of different rates of *Gaeumannomyces graminis* var. tritici inoculum for detecting take-all suppression in soils. Australas. Plant Pathol. 42, 103–109. 10.1007/s13313-012-0166-y

[B10] Chng S. Cromey M. G. Dodd S. L. Stewart A. Butler R. C. Jaspers M. V. (2015). Take-all decline in New Zealand wheat soils and the microorganisms associated with the potential mechanisms of disease suppression. Plant Soil. 397, 239–259. 10.1007/s11104-015-2620-4

[B11] Clapp R. A. (1998). Regions of refuge and the agrarian question: peasant agriculture and plantation forestry in Chilean Araucania. World Dev. 26, 571–589. 10.1016/S0305-750X(98)00010-2

[B12] Clarke K. R. (1993). Non-parametric multivariate analyses of changes in community structure. Aust. J. Ecol. 18, 117–143. 10.1111/j.1442-9993.1993.tb00438.x

[B13] Cook R. J. (2003). Take-all of wheat. Physiol. Mol. Plant Pathol. 62, 73–86. 10.1016/S0885-5765(03)00042-0

[B14] De Coninck B. Timmermans P. Vos C. Cammue B. P. A. Kazan K. (2015). What lies beneath: belowground defense strategies in plants. Trends Plant Sci. 20, 91–101. 10.1016/j.tplants.2014.09.007 25307784

[B15] de Ridder-Duine A. S. Kowalchuk G. A. Gunnewiek P. J. A. K. Smant W. Van Veen J. A. de Boer W. (2005). Rhizosphere bacterial community composition in natural stands of *Carex arenaria* (sand sedge) is determined by bulk soil community composition. Soil Biol. Biochem. 37, 349–357. 10.1016/j.soilbio.2004.08.005

[B16] Doughari J. (2015). An overview of plant immunity. J. Plant Pathol. Microbiol. 6:11 10.4172/2157-7471.1000322

[B17] Durán P. Acuña J. J. Jorquera M. A. Azcón R. Paredes C. Rengel Z. (2014). Endophytic bacteria from selenium-supplemented wheat plants could be useful for plant-growth promotion, biofortification and *Gaeumannomyces graminis* biocontrol in wheat production. Biol. Fertil. Soils 50, 983–990. 10.1007/s00374-014-0920-0

[B18] Fouly M. Wilkinson T. (2000). Detection of *Gaeumannomyces graminis* varieties using polymerase chain reaction with variety-specific primers. Plant Dis. 84, 947–951. 10.1094/PDIS.2000.84.9.947 30832025

[B19] Freeman J. Ward E. (2004). *Gaeumannomyces graminis*, the take-all fungus and its relatives. Mol. Plant Pathol. 5, 235–252. 10.1111/j.1364-3703.2004.00226.x 20565593

[B20] French S. Levy-Booth D. Samarajeewa A. Shannon K. E. Smith J. Trevors J. T. (2009). Elevated temperatures and carbon dioxide concentrations: effects on selected microbial activities in temperate agricultural soils. World J. Microbiol. Biotechnol. 25, 1887–1900. 10.1007/s11274-009-0107-2

[B21] Gardener B. B. M. Mavrodi D. V. Thomashow L. S. Weller D. M. (2001). A rapid polymerase chain reaction-based assay characterizing rhizosphere populations of 2.4-diacetylphloroglucinol-producing bacteria. Phytopathology 31, 44–54. 10.1094/PHYTO.2001.91.1.44 18944277

[B22] Glick B. R. (2015). Beneficial Plant-Bacterial Interactions. London: Springer.

[B23] Imperiali N. Dennert F. Schneider J. Laessle T. Mavrodi D. Maurhofer M.. (2017). Relationships between root pathogen resistance, abundance and expression of Pseudomonas antimicrobial genes, and soil properties in representative swiss agricultural soils. Front. Plant Sci. 8:427. 10.3389/fpls.2017.00427 28424714 PMC5372754

[B24] Iwamoto T. Tani K. Nakamura K. Suzuki E. Kitagawa M. Eguchi M.. (2000). Monitoring impact of *in situ* biostimulation treatment on groundwater bacterial community by DGGE. FEMS Microbiol. Ecol. 32, 129–141. 10.1111/j.1574-6941.2000.tb00707.x 10817866

[B25] Jara D. Herman A. Elizondo M. Ernesto A. (2011). Root disease suppressive soils: “Take all decline (*Gaeumannomyces graminis* var. *tritici*) in wheat. A. Agro Sur. 39, 67–78. 10.4206/agrosur.2011.v39n2-01

[B26] Jorquera M. A. Marileo L. G. Acuna J. J. Saggar S. (2014). Effect of nitrogen and phosphorus fertilization on the composition of rhizobacterial communities of two Chilean Andisol pastures. World J. Microbiol. Biotechnol. 30, 99–107. 10.1007/s11274-013-1427-9 23842756

[B27] Kwak Y.-S. Bonsall R. F. Okubara P. A. Paulitz T. C. Thomashow L. S. Weller D. M. (2012). Factors impacting the activity of 2,4-diacetylphloroglucinol-producing *Pseudomonas fluorescens* against take-all of wheat. Soil Biol. Biochem. 54, 48–56. 10.1016/j.soilbio.2012.05.012

[B28] Lee C. H. Barbier B. Bottos E. McDonald I. Craig-Cary S. (2012). The inter-valley soil comparative survey: the ecology of dry valley edaphic microbial communities. ISME J. 6, 1046–1057. 10.1038/ismej.2011.170 22170424 PMC3329106

[B29] Li L. Fan F. Song A. Yin C. Cui P. Li Z. (2017). Microbial composition and diversity are associated with plant performance: a case study on long-term fertilization effect on wheat growth in an Ultisol. Appl. Microb. Cell Physiol. 101, 4669–4681. 10.1007/s00253-017-8147-2 28188339

[B30] Liu B. Huang L. Kang Z. Buchenauer E. (2011). Evaluation of endophytic bacterial strains as antagonists of take-all in wheat caused by *Gaeumannomyces graminis* var. tritici in greenhouse and field. J. Pest. Sci. 84, 257–264. 10.1007/s10340-011-0355-4

[B31] Macia-Vicente J. G. Janson H.-B. Mendgen K. Lopez-llorca L. V. (2008). Colonization of barley roots by endophytic fungi and their reduction of take-all caused by *Gaeumannomyces graminis* var. tritici. Can. J. Microb. 609, 600–609. 10.1139/W08-047 18772922

[B32] Manici L. M. Bregaglio S. Fumagalli D. Donatelli M. (2014). Modelling soil borne fungal pathogens of arable crops under climate change. Int. J. Biometeorol. 58, 2071–2083. 10.1007/s00484-014-0808-6 24615638

[B33] Mavrodi D. V. Mavrodi O. V. Parejko J. A. Bonsall R. F. Kwak Y. S. Paulitz T. C.. (2012). Accumulation of the antibiotic phenazine- 1-carboxylic acid in the rhizosphere of dryland cereals. Appl. Environ. Microbiol. 78, 804–812. 10.1128/AEM.06784-11 22138981 PMC3264129

[B34] McSpadden Gardener B. B. Weller D. M. (2001). Changes in populations of rhizosphere bacteria associated with take-all disease of wheat. Appl. Environ. Microbiol. 67, 4414–4425. 10.1128/AEM.67.10.4414-4425.2001 11571137 PMC93184

[B35] Mendes R. Garbeva P. Raaijmakers J. M. (2013). The rhizosphere microbiome: significance of plant beneficial, plant pathogenic, and human pathogenic microorganisms. FEMS Microbiol. Rev. 37, 634–663. 10.1111/1574-6976.12028 23790204

[B36] Mendes R. Kruijt M. de Bruijn I. (2011). Deciphering the rhizosphere microbiome for disease-suppressive bacteria. Science 332, 1097–1100. 10.1126/science.1203980 21551032

[B37] Meza F. Silva D. (2009). Dynamic adaptation of maize and wheat production to climate change. Clim. Change 94, 143–156. 10.1007/s10584-009-9544-z

[B38] Montalba-Navarro R. (2004). Transformación de los agroecosistemas y degradación de los recursos naturales en el territorio mapuche: una aproximación histórico- ecológica. CUHSO 8, 19–40. 10.7770/cuhso-V8N1-art201

[B39] Mora M. L. Alfaro M. A. Jarvis S. C. Demanet R. Cartes P. (2006). Soil aluminium availability in Andisols of southern Chile and its effect on forage production and animal metabolism. Soil Use Manage. 22, 95–101. 10.1111/j.1475-2743.2006.00011.x

[B40] Mora L. Demanet R. Acuña J. J. Viscardi S. Jorquera M. Rengel Z. (2017). Aluminum-tolerant bacteria improve the plant growth and phosphorus content in ryegrass grown in a volcanic soil amended with cattle dung manure. Appl. Soil Ecol. 115, 19–26. 10.1016/j.apsoil.2017.03.013

[B41] Moya-Elizondo E. Arismendi N. Castro M. P. Doussoulin H. (2015).Distribution and prevalence of crown rot pathogens affecting wheat crops in southern Chile. Chilean J. Agric. Res. 75, 78–84. 10.4067/S0718-58392015000100011

[B42] Murphy J. Riley J. P. (1962). A modified single solution method for the determination of phosphate in natural waters. Anal. Chim. Acta 27, 31–36. 10.1016/S0003-2670(00)88444-5

[B43] Neuenschwander A. (2010). El Cambio Climático en el Sector Silvoagropecuario de Chile. Santiago: Salviat, S. A.

[B44] Nish M. (1973). Survival of *Gaeumannomyces graminis* var tritici in field soil stored in controled environments. Aust. J. Biol. Sci. 27, 2143–2145.

[B45] ODEPA, (2016). Oficina de Estudios y Políticas Agrarias. ODEPA, Santiago, Chile. Available online at: http://www.odepa.cl/rubro/cereales/ (Accessed March 2017).

[B46] Osbourn A. E. Clarke B. R. Lunness P. Scott P. R. Daniels M. J. (1994). An oat species lacking avenacin is susceptible to infection by *Gaeumannomyces graminis* var. tritici. Physiol. Mol. Plant Pathol. 45, 457–467. 10.1016/S0885-5765(05)80042-6

[B47] Perez C. A. Aravena J. C. Silva W. A. McCulloch R. Armesto J. J. Parfitt R. (2016). Patterns of ecosystem development in glacial foreland chronosequences: a comparative analysis of Chile and New Zealand. N.Z. J. Bot. 54, 156–174. 10.1080/0028825X.2016.1143018

[B48] Sagar R. Sharma G. (2012). Measurement of alpha diversity using Simpson index (1/λ): the jeopardy. Environ. Skep. Crit. 1, 23–24.

[B49] Shipton P. J. Cook R. J. Sitton J. W. (1973). Occurrence and transfer of a biological factor in soil that suppresses take-all of wheat in eastern Washington. Phytopathology 63, 511–517.

[B50] Silva S. Henriques M. Martins A. Oliveira R. Williams D. Azeredo J. (2009). Biofilms of non-*Candida albicans Candida* species: quantification, structure and matrix composition. Med. Mycol. 47, 681–689. 10.3109/13693780802549594 19888800

[B51] Smalla K. Oros-Sichler M. Milling A. Heuer E. Baumgarte S. Becker R.. (2007). Bacterial diversity of soils assessed by DGGE, T-RFLP and SSCP fingerprints of PCR-amplified 16S rRNA gene fragments: do the different methods provide similar results? J. Microb. Methods 69, 470–479. 10.1016/j.mimet.2007.02.014 17407797

[B52] Soussana J. F. Graux A. I. Tubiello F. N. (2010). Improving the use of modelling for projections of climate change impacts on crops and pastures. J. Exp. Bot. 61, 2217–2228. 10.1093/jxb/erq100 20410317

[B53] Taylor G. J. Foyd C. D. (1985). Mechanisms of aluminium tolerance in *Triticum aestivum* (wheat). II. Differential pH induced by winter cultivars in nutrient solutions. Am. J. Bot. 72, 695–701. 10.2307/2443681

[B54] Walkley A. Black I. A. (1934). An examination of Degtjareff method for determining soil organic matter and a proposed modification of the chromic acid titration method. Soil Sci. 37, 29–37. 10.1097/00010694-193401000-00003

[B55] Warncke D. Brown J. R. (1998). Potassium and other basic cations, in Recommended Chemical Soil Test Procedures for the North Central Region NCR Publication No. 221, ed Brown J. R. (Columbia, MO: Missouri Agricultural Experiment Station), 31–33.

[B56] Weller D. M. Mavrodi D. V. van Pelt J. A. Pieterse C. M. van Loon L. C. Bakker P. A. (2012). Induced systemic resistance in *Arabidopsis thaliana* against *Pseudomonas syringae* pv. tomato by 2,4-diacetylphloroglucinol-producing *Pseudomonas fluorescens*. Phytopathology 102, 403–412. 10.1094/PHYTO-08-11-0222 22409433

[B57] Weller D. M. Raaijmakers J. M. Gardener B. B. M. Thomashow L. S. (2002). Microbial populations responsible for specific soil suppressiveness to plant pathogens. Annu. Rev. Phytopathol. 40, 309–334. 10.1146/annurev.phyto.40.030402.110010 12147763

