# Supplementary material for: Screening and Characterization of Potentially Suppressive Soils against Gaeumannomyces graminis under Extensive Wheat Cropping by Chilean Indigenous Communities
Source: Front Microbiol. 2017 Aug 15;8:1552. doi: 10.3389/fmicb.2017.01552 (PMC5559505; doi:10.3389/fmicb.2017.01552)
Supplement: Supplementary file 1 [file DataSheet1.DOCX]

Supplementary Material

**Screening and characterization of potentially suppressive soils against *Gaeumannomyces graminis* under extensive wheat cropping by Chilean indigenous communities**

Paola Durán^*^, Milko Jorquera, Sharon Viscardi, Victor J Carrion, María de la Luz Mora and María J. Pozo^*^

* Corresponding authors:

Dr. Paola Durán: paola.duran@ufrontera.cl

Dr. María José Pozo: mjpozo@eez.csic.es

1. Supplementary Figures

**Sup. Fig. 1.** Schematic representation of *in vitro* plate dish (a) and tube (b) fungal inhibition assay.

**Sup. Fig. 2.** Biodiversity indexes Shanon-Wieneer (H), Simpson (represented by 1- λ) and S (species number) of bulk and rhizosphere soil samples. Tukey test was used to compare treatments means, values followed by the same letter do not differ at P < 0.05 (n= 5).

**Sup. Fig. 3.**  PCR amplification of ITS specific region from *Gaeumannomyces graminis* var tritici (Ggt) in 16 rhizosphere soils used in this study. C(+): positive control (Ggt), C(-): negative control (*Aspergillus niger*).

**Sup. Fig. 4.**  PCR amplification of ITS specific region of *Gaeumannomyces graminis* var tritici (Ggt) in plant tissues from inoculation assay in greenhouse of roots of Ggt infected wheat plants, Ggt (positive control), roots of uninfected wheat plants and *Aspergillus niger* (negative control)

**Sup. Fig. 5.**  PCR amplification of the PhlD gene for the detection of 2,4 DAPG producer bacteria in the rhizosphere soils used in the greenhouse assay. *Pseudomonas* sp. (SA 32A) and *Enterobacter* sp. (RJAL6) strains were used as positive c(+) and negative control c(-), respectively.
